# The Treatment of Cardio-Vascular Renal Disease

**Published:** 1915-12

**Authors:** Charles G. Stockton

**Affiliations:** Buffalo


### BUFFALO MEDICAL JOURNAL

Yearly Volume 71. DECEMBER, 1915. No. 5

### ORIGINAL ARTICLES

**The right is reserved to decline papers not dealing with practical medical and surgical subjects, and such as might offend or fail to interest readers. Contributors are solely responsible for opinions, methods of expression and revision of proof.**

### The Treatment of Cardio-Vascular Renal Disease.

By CHARLES G. STOCKTON, M. D., Buffalo.

Of the various types of cardio-vascular renal diseases I shall speak of but three, with special reference to treatment.

First—There is a type of valvular heart disease with myocardial changes, secondary renal disease and, later, vascular sclerosis. In the beginning there is low blood pressure and cyanotic kidneys. After a time there is renal insufficiency and true nephritis. A moderate uremia develops and there is an attempt towards high blood pressure which often is frustrated because the heart will not endure the increased effort. As the arterial tension rises, the strength of the heart declines and, with the dropping of blood pressure, the kidneys become more and more insufficient.

In these cases the treatment should be directed chiefly towards the heart. By improving the function of this organ, the vicious circle may be interrupted. Rest in bed is required, systematic massage, Neuheim baths, a limited intake of fluids, a diet moderate in amount and mixed in variety. Digitalis and its congeners are usually indicated and apocynum is called for, especially with the onset of edema.

Second—There is a form of diffuse nephritis, with the interstitial change dominant, more often seen in young adults, which may start from tonsillar or other infection. In some cases the etiology cannot be traced. The disease is characterized by great lowering of kidney function, accompanied by rise in blood pressure and cardiac hypertrophy and, at first, without obvious change in blood vessels except the element of arterial hypertension. The pathology seems to be primarily renal, the cardio-vascular condition coming as a result of kidney disease. When the arterial hypertension is relieved, the arteries may be scarcely palpable. Doubtless more striking evidences of vascular disease would appear if the disease was long continued. However, this arterial sclerosis is not often observed as the disease is relatively rapid in its course. Naturally, under the circumstances, there is a high degree of retention in the blood of uric acid, urea nitrogen and total nitrogen, especially of the total nitrogen. As a result of the intoxication, the patient suffers from precordial distress, cardiac arrhythmia and transient or persistent dyspnoea. There is retinitis, albuminuria and casts. The functional renal power is low. By stimulating the heart and fasting the patient temporary improvement occurs, perhaps to be followed by a sudden drop in systolic pressure, while the diastolic pressure remains high, a coincident rapid increase in nitrogen retention, the onset of a terminal coma.

This disease differs radically from the usual type of chronic interstitial nephritis which, as is well known, may endure for years with but moderate disturbance of the general health, so long as the patient's condition is carefully guarded. On the contrary, these cases in younger patients often endure but for a few months and are fatal.

By treatment the end may be delayed and the disease converted, perhaps, into a slower process. To be of real service, the treatment should be early begun and should keep the question of kidney function in the foreground. It is a fatal mistake to delay early radical treatment. The patient should rest in bed, the skin be stimulated with baths, and stagnation in the alimentary tract should be avoided.

The diet is of prime importance. The caloric intake should be so lowered that nitrogen retention in the blood is reduced. To accomplish this occasional starvation days may be required, the nitrogenous foods kept below fifty grammes per diem, and the total intake of food not above one thousand calories. In this matter patients vary considerably, both as to the retention and as to the effect of diet upon that retention.

Acidosis may be very high, but occasionally, or at least, temporarily, this is not the case. In one instance, seen with Dr. Allen Jones, the hydrogen ion concentration of the urine was found to be at six by the method of Sorenson*, the hydrogen ion concentration ofthe blood was found to be 7.6 by the method of Rovontree and Levi**, and the carbon dioxide tension of expired air was found to be 5.5 by the method of Fredrica***, the titratable acidity of the urine was 10, all evidences of the absence of acidosis. Nevertheless the total nitrogen

*Archiv. Expr. Path and Pharm. Indicator Method, alizerin, neutral red, phenolphalatein.
**Archiv. Int. Med., Oct. 1914.
***Berlin Klin. Woch., July 1914.

retention was 144 when it should have been 23 to 25, the urea nitrogen 72.6 instead of the normal 12 to 14, and the uric acid retention 11.9 instead of the usual one-half or one milligramme to one hundred cubic centimetres of blood. This high retention, with absence of acidosis, was to me a great surprise. Latterly, in all cases of nephritis showing acidosis, I have introduced Fischer's solution by the Murphy drip method, according to the full tolerance of the patient and, in emergencies, by the intravenous method. I have satisfied myself that the procedure is valuable. The taking of alkaline salts accomplishes but little in severe cases. It is not always easy to decide the question as to the amount of water to be permitted the patient. When the heart is not seriously involved, and the acidosis is high, I attempt to introduce an abundance of alkaline water, and for this purpose I have a high regard for the Source du Pavillon of Contrexeville and the Grande Source of Vittel. A colleague seems to have had success by repeated venesections, periods of fasting and the drinking of abundance of water, iso-tonic with the blood.

Third—There is a familiar type of cardio-vascular disease, usually seen in those just beyond middle age, more often in business men overtaxed with responsibility, men who eat too much, perhaps drink alcohol regularly, who have exercised too little and who, consequently, suffer from depression in the power to metabolize. The disease appears to rest on a failure in metabolism and a consequent auto-intoxication; yet it is probable that an arterio-sclerosis anticipates the loss in metabolic power in most cases.

His state of health may first attract the patient's attention because of attacks of dyspnoea or angina; however, his friends had noticed that he was becoming irritable and less efficient.

On effort at first, later without effort, while quiet in bed, there are attacks of shortness of breath, sometimes of asthma. There is complaint of mental confusion, **muscae volitantes,** tinnitus or other symptoms of cerebral disturbance. On examination there is found a high tension pulse, full arteries, slowly moving blood mass, high blood pressure, a trace of albumin in the urine, a few tube casts and the functional test shows renal insufficiency.

The heart is hypertrophied, often with gallop rhythm, signs of aortic roughening; later there is dilatation and mitral, perhaps tricuspid, insufficiency. As a rule there is a degree of pulmonary vesicular emphysema. The liver is enlarged and over sensitive to pressure, and functionally deficient. Edema develops in the dependent portions and soon there is evident anasarca, often with fluid in the serous cavities. Should the patient continue to satisfy his appetite, especially if he is permitted to select food according to his taste, his symptoms will become intensified and the physical signs will betray a steady advance in cardiac, arterial, hepatic and renal disease.

I have studied and directed the treatment of numerous cases of this sort in which I have had the good fortune to have the valuable laboratory co-operation of Dr. John L. Butsch. Invariably the metabolism has been found to be seriously compromised and acidosis has been shown to be an important factor.

As to metabolism, there is especially found a low nitrogen balance, often also a lowered carbohydrate balance, occasionally glycosuria. Invariably there is abnormal retention, not infrequently of high degree. This retention in one group of cases relates especially to uric acid. In ordinary health we rarely find more than 1.50 milligrammes of uric acid in 100 centigrammes of blood; more often not over .50 milligrammes, while in cases of this group we have found 5.00 milligrammes to 10.00 milligrammes or more, and yet without manifestations of gout.

In another group of cases the retention in the blood relates especially to urea nitrogen and total nitrogen, which may rise to a point two, three or four times the normal.

In yet another group of cases the retention in the blood is very general, including not only a high percentage of uric acid, but also nitrogen, urea and also glucose.

We have interpreted these findings as meaning, in the uric acid group, that the liver was specially incompetent; in the nitrogen retention group, that the kidneys were particularly defective; in the group showing retention in a wide sense, that the whole metabolic and eliminative mechanism was crippled.

The clinical study of these cases confirmed the conclusion reached by laboratory investigation. In all groups the cardiac involvement was judged to average about the same; in the uric acid cases, there were evidences of hepatic insufficiency and all the symptoms of uricacidemia; in the nitrogen retention group, there was found, by the phenolsulphonephthalien test, a poor renal function, and there were the symptoms of uremia, often in excess of the cardio-vascular symptoms, grave though they might be.

All these observations were of great interest to us, but the interest was intensified by finding that by methods of diet, exercise, baths and alkalinisation we were enabled to relieve these cases in a manner more satisfactory than we had noted by any other methods of treatment. It is true that resort was had to digitalis, to apocynum and, occasionally, to the nitrites or to adrenaline, as the case might indicate. The point is that the improvement observed was over and beyond that which these drugs alone would induce; further, it usually became possible to abandon early the use of these drugs altogether, the patients gaining steadily so long as the dietetic and other hygienic measures were strictly followed, governing the intake of nitrogenous, carbohydrate and fatty foods by the studies of the blood and urine, weighing carefully the food, and estimating the caloric value thereof.

In the beginning starvation days are prescribed, much as is adopted in diabetic cases; following this we commonly resort to the Karell diet, made known in this country by the publication of Dr. Freeman Ford Ward* of New York. This consists in allowing the patient 200 c.c. of milk at four hours' interval, four times in twenty-four hours, permitting no water or other fluids save the milk. Generally speaking, the urine at once rises in amount, so that from two to five times as much urine is discharged as there has been fluid imbibed in the twenty-four hours. The edema promptly subsides together with the dyspnoea, precordial distress and other symptoms. This limited milk diet may be pursued for a few days or even a week, after which an increase is made in food and drink, according to indication. We are careful not to prolong starvation so long that the patient's vitality is lowered. We gradually increase the intake, but govern the quantity by study of the metabolism, never permitting the patient to exceed his nitrogen or carbohydrate balance.

To illustrate, Mr. B— came to me five years ago, suffering from attacks of dyspnoea, angina, precordial and cerebral distress, congested lungs, large liver, scanty, albuminous urine, containing numerous casts and blood cells, having general edema, apparently approaching his end. He was not syphilitic. He is a large and intelligent man, overworked in business, a big eater and formerly fond of his "highballs." His nitrogen and carbohydrate balance was determined for me by my late lamented colleague, Dr. Fred. C. Busch. The patient's dietary was arranged according to his metabolic possibilities. He scrupulously obeyed his instructions and at this day he feels perfectly well. His once enormous heart is much smaller, he escapes dyspnoea, angina and edema. However, the urine continues to show albumin and casts, and, by the functional test, he discharges only 19% of the phenolsulphonephthalein in two hours. He has still the mitral insufficiency. Whereas his systolic blood pressure was formerly about 200, it is now 170 systolic, 120 diastolic, and the heart load 50. He is now taking about 1894 calories: proteid 39.50 gm., carbohydrate 186 gm., fats 79 gm. He voids 1014 c.c. of urine, containing 2 grammes of albumin. The acidity is 46 by tit-

---

*Medical Record, 1912, Mar. 30.

ration. He discharges 35.8 gm. total solids and 13.20 gm. of urea. Ammonia, .436 gm., hydrogen ion concentration in urine 5.7, the normal being 6. Hydrogen ion concentration in the blood is 7.4, this being the low normal. The $CO^2$ in alveolar air is 4.9%, the normal being 5 to 6%. The percentage of $CO^2$ in alveolar air indicates in this case a slight degree of acidosis. The hydrogen ion concentration in the blood corresponds, but the urine shows no acidosis.

You will remark that this does not sound like a very well man. Quite true; he is a very much diseased man; his organs were badly and permanently damaged when I first saw him. But this is the point: this seriously ill man, 64 years old, has been symptom-free for all these years; he is living and happy, simply because he cheerfully submits to and never oversteps certain strict limitations of living that have been imposed. I wonder from year to year how long he is to continue. His case is the longest, yet I have another nearly parallel and several others that approach these.

I have spoken of the use of alkalies in these cases. That brings up another question, that of acidosis. At one time I supposed that acidosis was controllable by the administration of indifferent alkalies by the mouth. The studies of Palmer, Henderson and others have shown me that I was wrong. The titratable acidity of urine can be controlled by alkalies, certain symptoms are thus relieved; yet in a deeper sense acidosis is not relieved by ordinary alkaline drugs. It is a question of hydrogen ion concentration. This deeper acidosis (so to speak), if relieved, must be so by having recourse to dietetic measures, by increasing tissue oxygenation rather than by drugs. Nevertheless, certain alkalies are clinically of distinct value. And here again I must refer to the benefit which I have seen patients receive at the French spas, in the Vosges, near where the conflict is now raging; I refer to Contrexeville and Vittel. If time permitted I should like to report the favorable effect which during three different visits at these springs I have observed in retention cases, uric acid and nitrogen retention, cases analogous to those which I have described in this group.

It may be suggested that the improvement noted was the result of change in environment, to release from business cares and to relaxation. Of course, these factors are to be counted, but the action of the water cannot thus be dismissed.

There remains to be considered those cases that are syphilitic. No influence is more potent than this infection in crippling metabolic power. Often there is extensive and lasting damage to the parenchyma of important organs. However, there is usually a percentage of functional disability which can be removed by suitable specific medication.

With cases wherein the heart, arteries and kidneys are seriously compromised, great circumspection must be practised in using sufficient mercury and arsenic to accomplish real benefit. Some cases that seem very unpromising will make wonderful gain under Salvarsan and hydragyrum, and although a proportion of cases will be harmed and occasionally one will quickly succumb, I am accustomed to try specific medication guardedly and watchfully. One must be very careful where there is kidney involvement; and nearly as much danger exists in using effective doses in syphilis of the heart.

We have noted in certain luetic patients in whom there is very marked metabolic deficiency, a curious contradiction in the metabolic processes, from time to time in the course of the disease. This is sufficiently striking to lead to suspect syphilis even when the Wassermann reaction is negative. For instance, one case originally showed renal insufficiency, very high retention, especially of uric acid. There was acidosis and the hydrogen ion concentration proved it.

The patient was relieved by diet from edema, dyspnoea, and arrhythemia. After eighteen months with the same treatment, the dyspnoea and gallop rhythm returned. We suspected a return of acidosis, but on investigation we found a good functional renal power, a normal $CO^2$ tension and normal hydrogen ion concentration in blood and urine. There was a systolic blood pressure of about 200, a diastolic pressure of 125. There seemed to be no good reason for the return of the symptoms. Although there was a negative Wassermann test, we gave 18 cgm of Salvarsan and the patient was relieved of his symptoms, saying that he felt better than for two years. This man was made worse by the use of Fischer's solution as might have been expected. Fischer's solution is useful in kidney insufficiency with acidosis; it is harmful in edema and other symptoms that are cardio-vascular in origin.

---

**Disinfection of the Hands and Abdominal Skin Before Operation.** McDonald (Surgery, Gynecology and Obstetrics, July 1915) advises the following solution:

Acetone (commercial), 40 parts;
Denatured alcohol, 60 parts;
Pyxol, 2 parts.

With this solution, in no case was any growth obtained after thirty seconds' immersion. This period, however, is too short in practice, and one minute is advised to provide a margin of safety. Preliminary washing with soap and water was found to be no aid to efficiency, but was rather a slight hindrance. It had the additional disadvantage of irritating the hands.

